# CsZAT12 interacts with CsNAC87 to repress chlorophyll accumulation in albino leaves of tea plants

**DOI:** 10.1093/hr/uhag295

**Published:** 2026-07-09

**Authors:** Yafei Guo, Dan Yang, Ying Sun, Xin Cheng, Huanhuan Zong, Zhaoliang Zhang, Weiwei Deng, Xiaochun Wan, Linlin Liu, Qi Chen

**Affiliations:** National Key Laboratory for Tea Plant Germplasm Innovation and Resource Utilization, Anhui Agricultural University, Hefei 230036, China; National Key Laboratory for Tea Plant Germplasm Innovation and Resource Utilization, Anhui Agricultural University, Hefei 230036, China; National Key Laboratory for Tea Plant Germplasm Innovation and Resource Utilization, Anhui Agricultural University, Hefei 230036, China; National Key Laboratory for Tea Plant Germplasm Innovation and Resource Utilization, Anhui Agricultural University, Hefei 230036, China; National Key Laboratory for Tea Plant Germplasm Innovation and Resource Utilization, Anhui Agricultural University, Hefei 230036, China; National Key Laboratory for Tea Plant Germplasm Innovation and Resource Utilization, Anhui Agricultural University, Hefei 230036, China; National Key Laboratory for Tea Plant Germplasm Innovation and Resource Utilization, Anhui Agricultural University, Hefei 230036, China; National Key Laboratory for Tea Plant Germplasm Innovation and Resource Utilization, Anhui Agricultural University, Hefei 230036, China; National Key Laboratory for Tea Plant Germplasm Innovation and Resource Utilization, Anhui Agricultural University, Hefei 230036, China; National Key Laboratory for Tea Plant Germplasm Innovation and Resource Utilization, Anhui Agricultural University, Hefei 230036, China

## Abstract

The albino leaves of cold-sensitive tea plants exhibited a marked reduction in chlorophyll content. However, the underlying regulatory mechanism remains elusive. In this study, we determined lower chlorophyll levels in tender leaves of cold-sensitive ‘Baiye 1’ compared to green leaves during the spring. Comparative transcriptome analyses identified the chlorophyll biosynthesis gene *CsPORA*, which was significantly downregulated in albino leaves. Functional assays confirmed that *CsPORA* positively regulates chlorophyll content. Electrophoretic mobility shift assay, yeast one-hybrid, dual-luciferase, and GUS staining results demonstrated that CsNAC87 binds to the promoter of *CsPORA* and suppresses its expression. Additionally, CsZAT12 physically interacts with CsNAC87 to enhance the repression. Overexpression of either *CsZAT12* or *CsNAC87* negatively regulated chlorophyll accumulation in tea plants and tobacco leaves, while co-transformation of *CsZAT12* and *CsNAC87* intensified the albino phenotype in tobacco leaves. Low temperature treatment of ‘Baiye 1’ triggered the marked upregulation of *CsNAC87* and *CsZAT12*, leading to dramatical downregulation of *CsPORA* and leaf albinism. Collectively, our study reveals a novel cold-responsive CsZAT12-CsNAC87 module that negatively regulates chlorophyll synthesis by repressing *CsPORA*, providing new insights into chlorophyll metabolism in albino leaves of cold-sensitive tea cultivars.

## Introduction

Leaf coloration serves as a critical phenotypic trait that influences not only the growth, development, flavor, and quality of leaf-for-use plants, but also their marketability and consumer acceptance. Tea plant (*Camellia sinensis*) is one of the most important commercial leaf-for-use crops in the world. Tea leaves are processed into different tea products, and these products has been being popular all over the world. Therefore, the leaf color traits (albino leaves, purple leaves, yellow leaves, and variegated leaves) of tea plants have been extensively studied [1–4]. Among them, cold-sensitive albino tea cultivars, such as ‘Baiye 1’ (syn. ‘Anji Baicha’; [5, 6]), ‘Xiaoxueya’ [7], and ‘Huabai 1’ [8], have been characterized. Compared to green leaves, albino tea leaves exhibited distinct albinism, characterized by chlorophyll deficiency yet elevated accumulation of free amino acids [6, 9, 10]. However, the regulatory mechanism underlying chlorophyll deficiency in albino tea leaves is still largely unknown.

Chlorophyll is one of the most important photosynthetic pigments in plants. Chlorophyll homeostasis is not only the foundation for plants to convert photosynthesis into chemical energy, but also crucial for the growth and development [11]. The synthesis of chlorophyll is regulated by the substrate glutamic acid via a series of enzymatic reactions. POR encodes a protochlorophyllide oxidoreductase in the biosynthesis pathway of chlorophyll [12]. Loss of *CsPOR1* resulted in an albino phenotype in tea leaves [13]. The magnesium chelatase enzyme complex is composed of three subunits: CHLI, CHLD, and CHLH. Overexpression of *CsCHLI*, isolated from a yellow-leaved tea plant ‘Baijiguan’, in *Arabidopsis* resulted in a reduction in chlorophyll content [14]. *CsNYC1a* (*NON-YELLOW COLORING 1*), cloned from a yellow-leaved tea plant ‘Nanchuan Huangye 1’, overexpressed in *Arabidopsis* to inhibit the accumulation of chlorophyll [15]. *CsCLH1* (*Chlorophyllase*) overexpression reduced chlorophyll content in tea leaves [16].

The regulation of chlorophyll metabolism has been well characterized in diverse species. NAC (NAM, ATAF1/2, and CUC2) TFs play an important role in chlorophyll metabolism in plants. For instance, ANAC087 in *Arabidopsis* accelerated leaf aging by activating *PPH*, *PAO* and *NYE1* expression [17]. BrNAC087 positively regulated the expression of chlorophyll catabolic genes *BrPPH* and *BrRCCR*, inhibiting chlorophyll content in Chinese flowering cabbage [18]. FcrNAC22 negatively regulated chlorophyll content in Kumquat fruit via activating chlorophyll catabolic genes *FcrSGR*, *FcrCLH*, *FcrPPH*, *FcrPAO*, and *FcrNYC1* [19]. However, researches on NAC TFs that inhibit chlorophyll biosynthesis in plants are still largely unknown. The C2H2-type zinc finger proteins play an important role in regulating chlorophyll metabolism in plants. In tomato, SlZHD17 positively regulated *SlSGR1* to inhibit the accumulation of chlorophyll in tomato fruit [20]. *SlZAT5* overexpression induced the accumulation of chlorophyll in transgenic fruits to delay tomato fruit ripening [21]. MdZAT10 interacted with MdBT2 to positively regulate JA-induced leaf senescence in apple [22]. Environmental factors, such as low temperature, also affects chlorophyll homeostasis by affecting the activity of chlorophyll-related enzymes. For instance, cold-responsive SlZF61(C2H2) interacted with SlWRKY51 to affect chlorophyll synthesis in tomato [23]. Cold-inducible CsCBF1 accelerated chlorophyll catabolism via activating *CsSGR* expression in cold-sensitive cucumber [24]. In tea plants, cold-responsive CsABF2 negatively regulated chlorophyll accumulation via activating *CsSGR1* expression [25]. *CsGLK1/2* positively regulated chlorophyll level in transgenic tomato [26]. However, the role of NAC and ZAT TFs regulating the chlorophyll metabolism in tea plants is still unclear.

In this work, we determine chlorophyll content in tender leaves of the cold-sensitive tea cultivar ‘Baiye1’ across different periods during the spring. Comparative transcriptome analyses were conducted using the RNA-Seq data from albino and green leaves. *CsPORA* was found to be significantly downregulated in albino tea leaves, and its expression level showed a strong positive correlation with chlorophyll content. Transient overexpression and silencing assays confirmed that *CsPORA* positively regulates chlorophyll synthesis. Electrophoretic mobility shift assay (EMSA), yeast one-hybrid (Y1H), dual-luciferase reporter assay, and GUS staining assay were performed, and we confirmed that CsNAC87 negatively regulates *CsPORA*, thereby repressing chlorophyll biosynthesis. CsZAT12 physically interacts with CsNAC87 to strengthen the negative regulation of CsNAC87 on *CsPORA*. Co-expression of *CsZAT12* and *CsNAC87* in tobacco leaves leads to a noticeable albino phenotype. Moreover, low temperature significantly induced the expression of *CsZAT12* and *CsNAC87* but inhibited the expression of *CsPORA*, leading to leaf albinism in ‘Baiye 1’. Collectively, our findings offer novel insights into the regulatory mechanism underlying leaf albinism in cold-sensitive tea plants.

## Results

### Marked reduction of chlorophyll content in albino tea leaves

**Figure 1:**
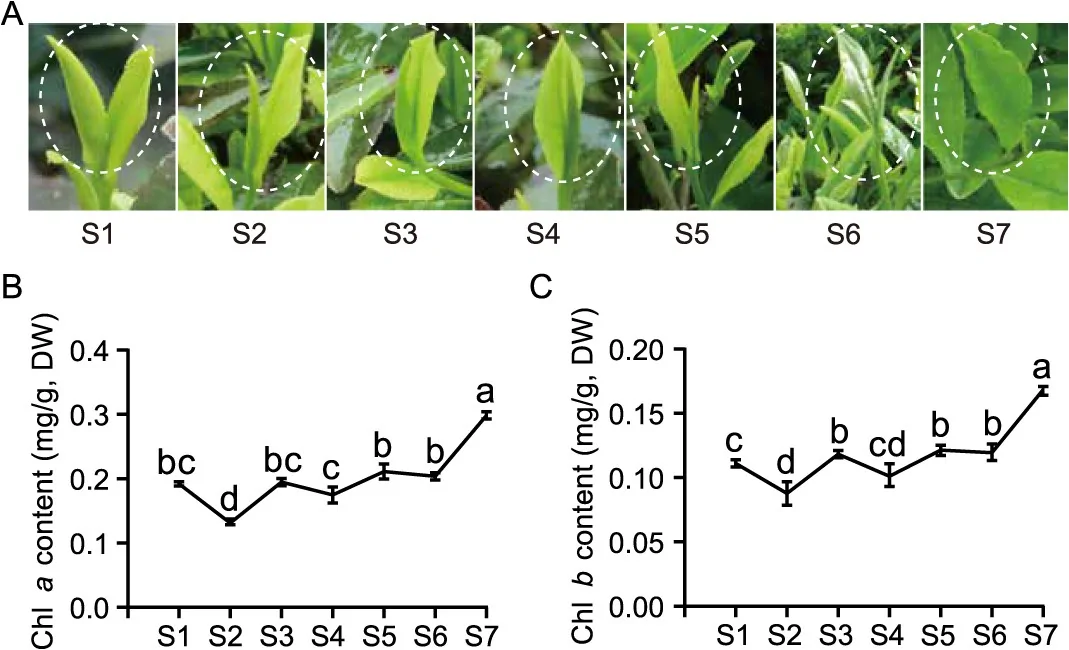
Analysis of chlorophyll content in tender leaves of ‘Baiye 1’ at different periods (S1-S7). (A) Characterization of representative field phenotypes in tender leaves of ‘Baiye 1’ tea cultivar across different periods during the spring. (B and C) The accumulation of Chl *a* and Chl *b* in tender leaves of ‘Baiye 1’ at different periods. DW, dried weight. S1, 28^th^ Mar; S2, 6^th^ Apr; S3, 12^th^ Apr; S4, 20^th^ Apr; S5, 27^th^ Apr; S6, 4^th^ May; S7, 16^th^ May. Different letters above line graphs indicate different significance groups (*P* < 0.05).

To investigate the differences in chlorophyll accumulation profiles of chlorophyll between green and albino tea leaves, we collected tender leaves of ‘Baiye 1’ at different periods during the spring season. In spring, tender leaves of ‘Baiye 1’ turned white from S1 to S2, then the leaf color gradually became green from S2 to S7 (Fig. 1A). Accordingly, the contents of chlorophyll *a* (Chl *a*) and chlorophyll *b* (Chl *b*) decreased from S1 to S2 and then increased from S2 to S7 (Fig. 1B and C). Accordingly, the effective accumulated temperature exhibited a positive correlation with the leaf color phenotype and chlorophyll content (Table S1; Fig. 1). These results suggest that chlorophyll content is lower in albino tea leaves than in green leaves, and that environmental temperature influences chlorophyll accumulation.

### Comparative transcriptome analyses reveal a significant reduction of *CsPORA* expression in albino tea leaves

**Figure 2:**
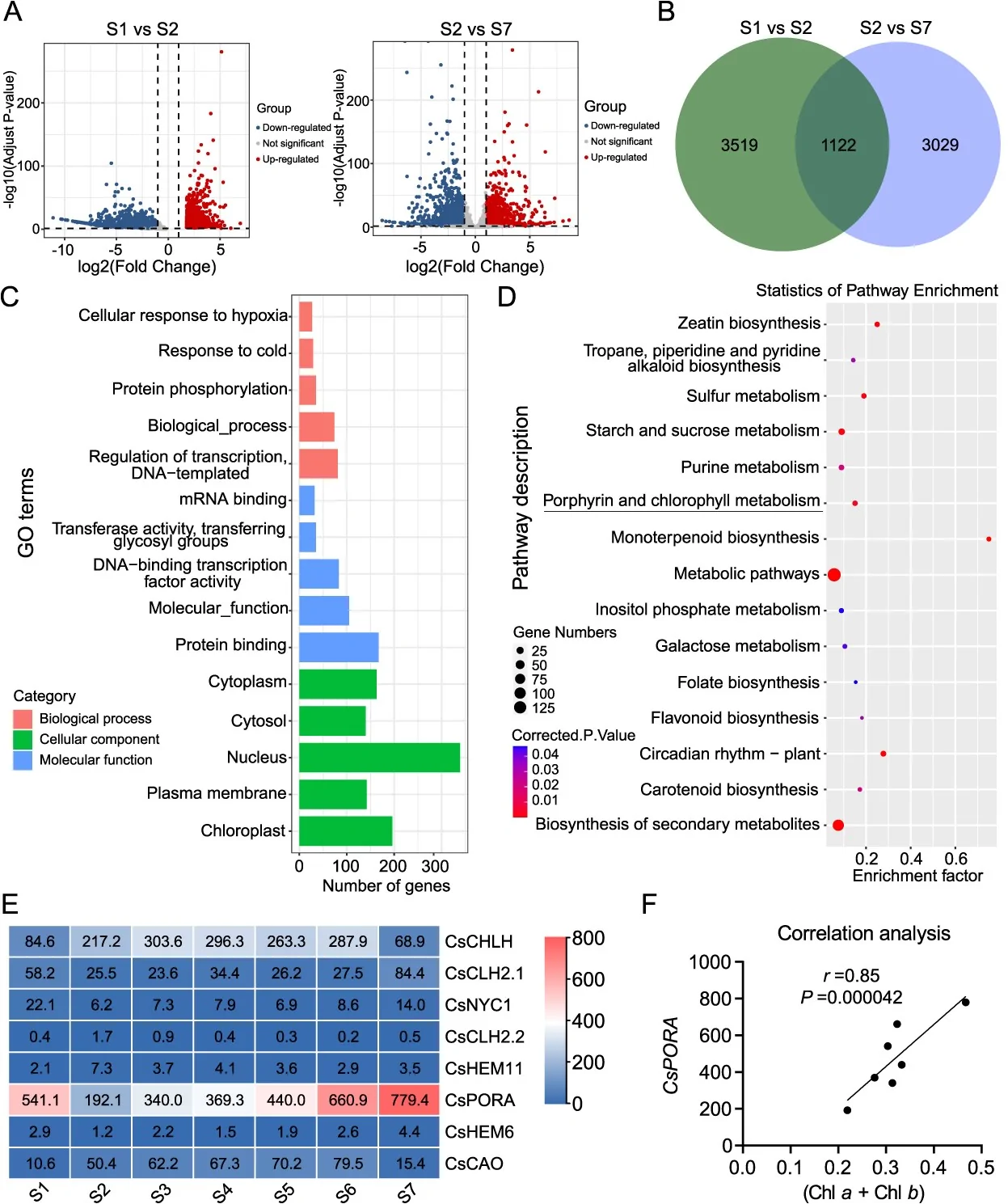
Comparative transcriptome analyses reveal the expression profiles of DEGs between albino and green leaves in tea plants. (A) Volcano plots analysis of DEGs in the comparisons of S1 vs S2 and S2 vs S7. (B) Venn plot analysis of DEGs shared between the group of S1 vs S2 and the group of S2 vs S7. (C and D) GO and KEGG enrichment analyses of DEGs. (E) The expression profile of DGEs identified in the item of ‘Porphyrin and chlorophyll metabolism’. The numbers in the box represent the expression levels of these genes. (F) Correlation analysis between the expression of *CsPORA* and chlorophyll content in tender leaves of ‘Baiye1’ across different periods.

To investigate the molecular basis of the differences in chlorophyll accumulation between albino and green leaves, we performed differentially expressed genes (DEG) analysis using transcriptome data of ‘Baiye 1’ at stages S1, S2, and S7 (S1 vs S2, S2 vs S7; |log_2_(Foldchange)| ≥ 1) (Fig. 2A). A total of 4641 (S1 vs S2) and 4151 DEGs (S2 vs S7) were identified, respectively. Among these two comparisons, a total of 1122 DEGs were commonly identified (Fig. 2B; Table S2), and these genes were used for Gene Ontology (GO) and KEGG enrichment analyses. GO results revealed that DEGs are significantly enriched in the pathways of ‘response to cold’, ‘protein phosphorylation’, and ‘regulation of transcription, DNA-templated’ (Fig. 2C). KEGG results showed that DEGs are primarily enriched in pathways of ‘Porphyrin and chlorophyll metabolism’, ‘Carotenoid biosynthesis’, ‘Purine metabolism’, ‘Flavonoid biosynthesis’, ‘Starch and sucrose metabolism’, ‘Biosynthesis of secondary metabolites’ and ‘Metabolic pathways’ (Fig. 2D). Eight DEGs associated with chlorophyll metabolism, namely *CsCHLH*, *CsCLH2*.*1*, *CsNYC1*, *CsCLH2.2*, *CsHEM11*, *CsPORA*, *CsHEM6*, and *CsCAO*, were identified in the term ‘Porphyrin and chlorophyll metabolism’ (Fig. 2E). Notably, the expression level of *CsPORA* was higher than those of the other seven genes (Fig. 2E). Moreover, the expression of *CsPORA* across stages S1-S7 exhibited a positive correlation (*r* = 0.85, *P* =0.000042) with chlorophyll content (Fig. 2F; Fig. S1). These results suggest that low temperature may induce the pronounced down-regulation of *CsPORA*, leading to impaired chlorophyll biosynthesis and thereby causing leaf albinism in ‘Baiye 1’.

### CsNAC87 negatively regulates *CsPORA* to repress chlorophyll synthesis

**Figure 3:**
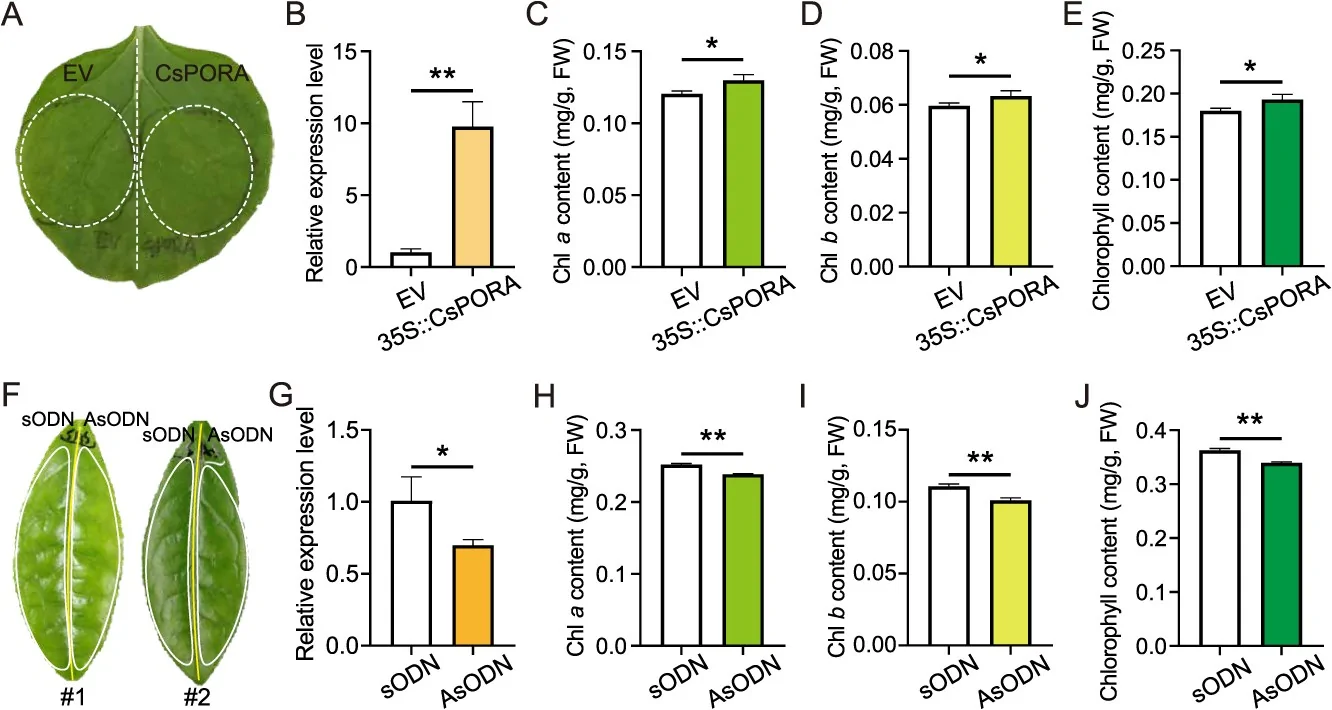
Functional analysis of *CsPORA* in regulating chlorophyll metabolism. (A and B) Leaf color phenotype and relative expression analysis of *CsPORA* in transgenic tobacco leaves. (C–E) Determination of Chl *a*, Chl *b*, and total chlorophyll contents in transgenic tobacco leaves of p1305 (EV) and p1305-CsPORA (35S::CsPORA). (F and G) Leaf color phenotype and relative expression analysis of *CsPORA* in AsODN-mediated tea leaves. (H–J) Analysis of the contents of Chl *a*, Chl *b*, and total chlorophyll in silenced tea leaves of *CsPORA*. FW, fresh weight.

To verify the role of *CsPORA* in chlorophyll biosynthesis, transient overexpression assays were performed. Overexpression of *CsPORA* in tobacco leaves significantly induced the accumulation of Chl *a*, Chl *b*, and total chlorophyll (Fig. 3A–E). In tea plants, silencing of *CsPORA* caused a decline of its expression and the contents of Chl *a*, Chl *b*, and total chlorophyll in tea leaves (Fig. 3F–J). These results indicate that *CsPORA* may play an important role in the biosynthesis of chlorophyll in tea plants.

**Figure 4:**
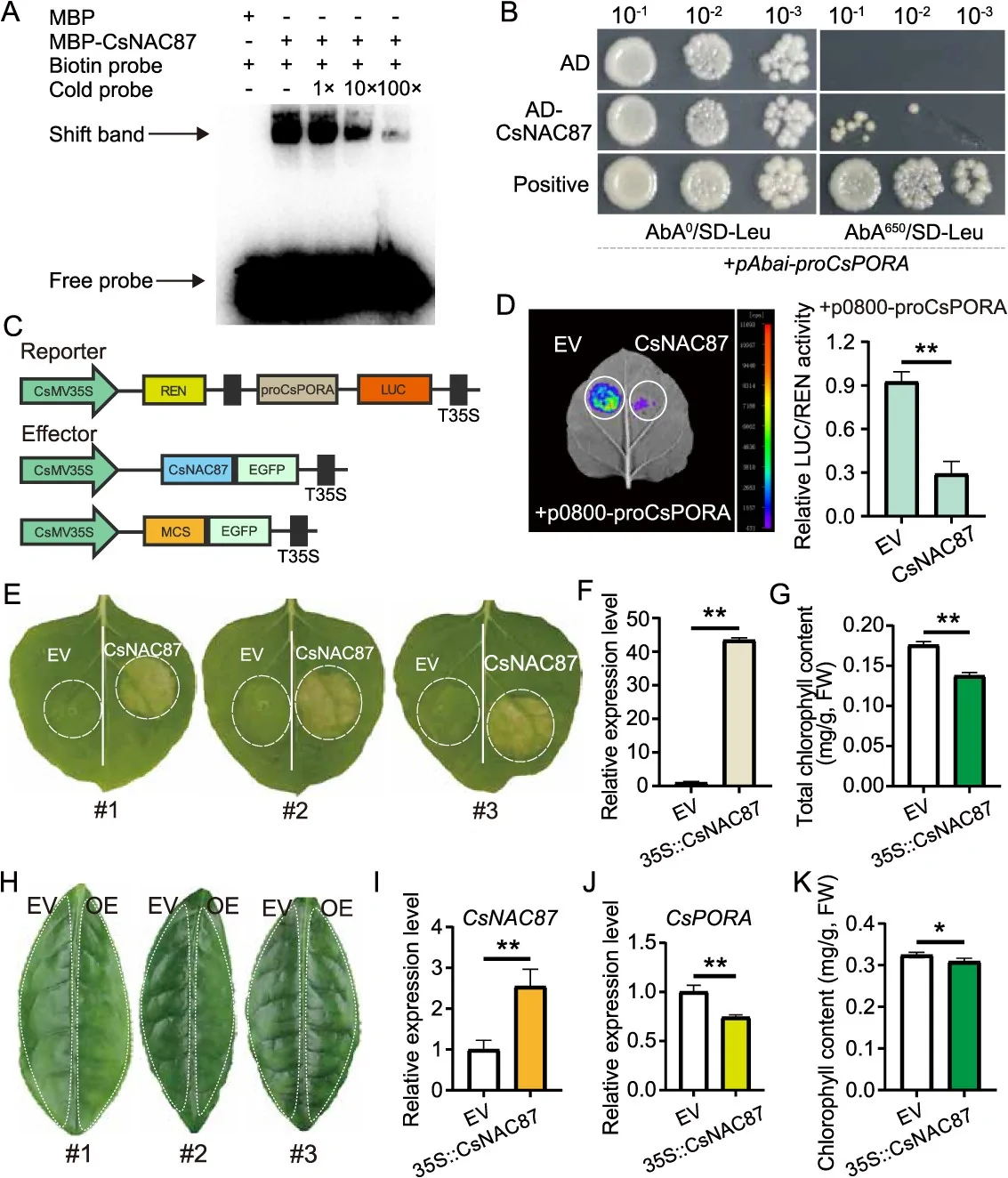
CsNAC87 negatively regulates *CsPORA* to inhibit the accumulation of chlorophyll. (A) EMSA shows the direct binding of CsNAC87 to the promoter of *CsPORA*. The un-labelled probe was used as competitor in 1-, 10- and 100-folds excess to the biotin-labelled probe. ‘+’ means addition, and ‘−’ means no addition. (B) Growth of yeast cells co-transformed with positive control, baits + prey or negative control on SD/−Leu medium added with or without AbA; AbA^650^ indicates SD/−Leu medium added with 650 μg/L AbA. (C) Schematic diagrams of the reporter and effectors in LUC assays; MCS, multiple cloning sites; T35S, CaMV 35S terminator. LUC, firefly luciferase; REN, Renilla luciferase. (D) Representative bioluminescence imaging of tobacco leaves infiltrated with the effector and p0800-proCsPORA and relative LUC/REN activity analyses. (E) The leaf coloration comparison of transgenic tobacco plants expressing p1305-CsNAC87 and the EV. (F) Relative expression of *CsNAC87* in transgenic tobacco leaves. (G) Determination of chlorophyll content in *CsNAC87*-overexpressing tobacco leaves. (H) Differences in leaf color phenotype between transgenic tea leaves of the EV and p1305-CsNAC87. (I and J) Relative expression of *CsNAC87* and *CsPORA* in transgenic tea leaves. (K) Chlorophyll content in transgenic tea leaves of EV and p1305-CsNAC87.

To investigate transcription factors that regulate chlorophyll metabolism through *CsPORA*, we conducted an in-depth analysis of transcription factors in both the S1 vs S2 and S2 vs S7 comparisons. Co-expression network analysis identified an NAC transcription factor CsNAC87, which exhibited a highly negative correlation with both *CsPORA* expression and chlorophyll content (Fig. S2A–C). MBP-CsNAC87 protein was purified *in vitro* (Fig. S3). EMSA results revealed that CsNAC87 could bind to the TTACTTGT motif within the promoter of *CsPORA* (Fig. 4A). Yeast one-hybrid assays further confirmed that CsNAC87 binds to the promoter of *CsPORA* (Fig. 4B). The results of dual-luciferase reporter assays revealed that CsNAC87 inhibits the expression of *CsPORA* (Fig. 4C and D). Collectively, above results indicate that CsNAC87 regulates chlorophyll metabolism by inhibiting *CsPORA* expression.

We further analyzed the role of CsNAC87 in regulating chlorophyll metabolism. *CsNAC87* was overexpressed in tobacco leaves, and transgenic tobacco leaves turned white (Fig. 4E and F). Chlorophyll content was significantly reduced compared with the control (Fig. 4G). Moreover, *CsNAC87* overexpression in tea leaves induced chlorosis compared with the control group (empty vector, EV) (Fig. 4H and I). The expression of *CsPORA* was reduced (Fig. 4J), and chlorophyll content also decreased markedly in *CsNAC87*-overexpressing tea leaves (Fig. 4K). These results indicate that CsNAC87 is an important repressor of chlorophyll accumulation in tea plants.

### CsZAT12 interacts with CsNAC87 to repress *CsPORA* expression

**Figure 5:**
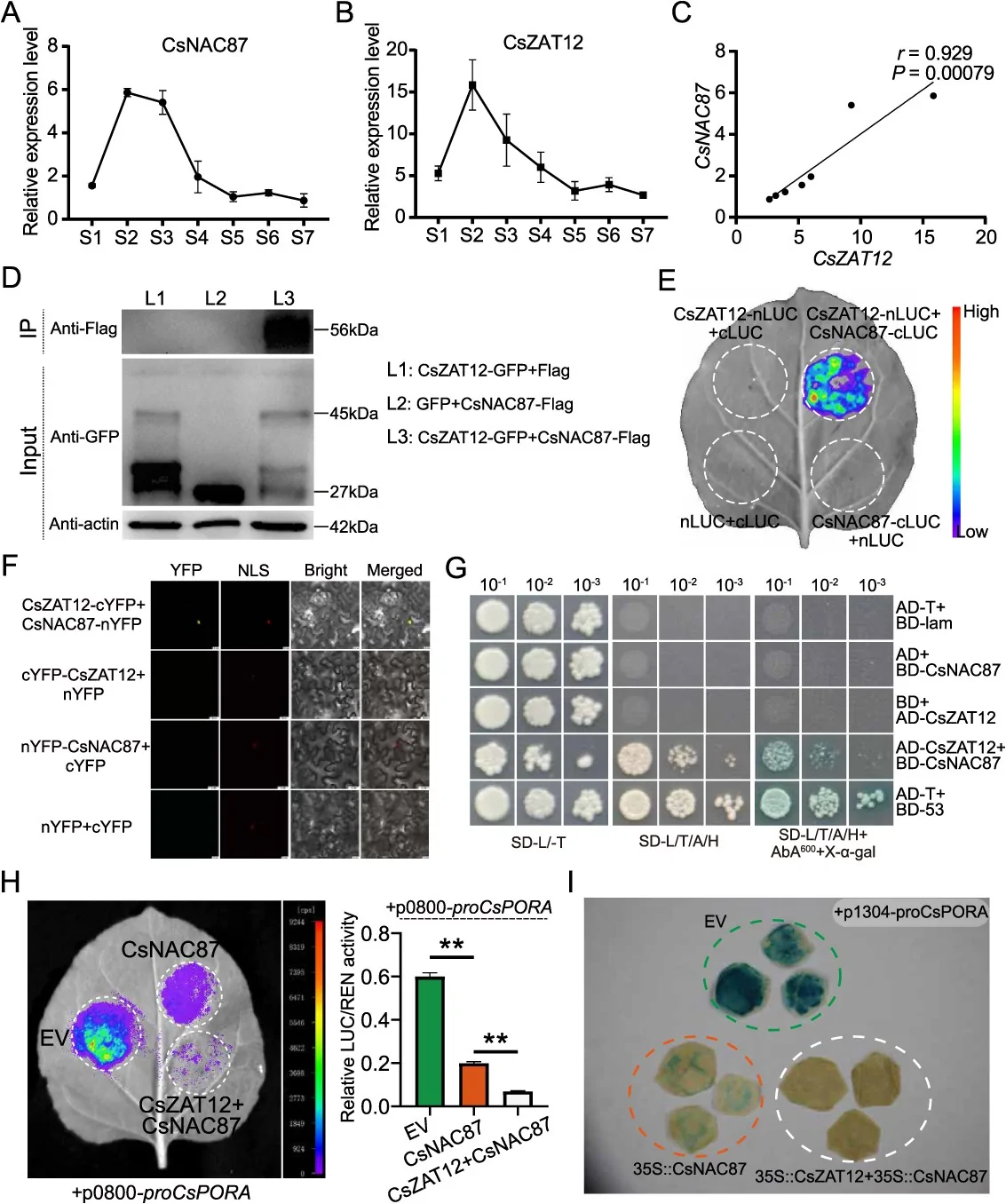
CsZAT12 physically interacts with CsNAC87 to repress *CsPORA* expression. (A and B) The expression profiles of *CsNAC87* and *CsZAT12* in tender leaves of ‘Baiye1’ across different periods. (C) Correlation analysis between the expression of *CsNAC87* and *CsZAT12* across different periods. (D) Co-IP analysis of the interaction between CsZAT12 and CsNAC87 in tobacco leaves. The molecular weights of CsZAT12-GFP, CsNAC87–3 × Flag, free GFP, and actin are 46 kDa, 56 kDa, 27 kDa, and 42 kDa, respectively. (E) LCI assays showing the interaction between CsZAT12 and CsNAC87. (F) BiFC assays for verifying the interaction between CsZAT12 and CsNAC87 in the nucleus. (G) Growth of yeast cells co-transformed with positive control (AD-T + BD-53), negative control (AD-T + BD-Lam) and AD-CsZAT12 + BD-CsNAC87 on SD/−Trp/−Leu medium and SD/−Trp/−Leu/-His/−Ade with or without X-α-gal. (H) Fluorescent imaging and Relative LUC/REN ratios of tobacco leaves infiltrated with CsNAC87 alone or with CsZAT12 and CsNAC87 together, and the reporter made using *CsPORA* promoter. (I) GUS staining analyses of CsNAC87 and the combination of CsZAT12 and CsNAC87 on the activity of *CsPORA* promoter.

Among the transcription factors present in the DEGs common to both the S1 vs. S2 and S2 vs. S7 comparisons, *CsZAT12*, encoding a C2H2-type zinc finger protein, was found to be highly correlated with *CsNAC87* expression across different stages (*r* = 0.929, *P* =0.00079; Fig. 5A–C). Co-IP assays were conducted (Fig. S4A–D), and the results demonstrated that CsZAT12 co-precipitated with CsNAC87 *in vivo* (Fig. 5D). This interaction was supported by luciferase complementation imaging (LCI) assays that luciferase (LUC) activity was strongly reconstituted in the tobacco leaves co-expressing CsZAT12-nLUC and CsNAC87-cLUC (Fig. 5E). Bimolecular fluorescence complementation (BiFC) assays showed that co-infiltration of CsZAT12-cYFP and CsNAC87-nYFP in tobacco leaves led to recovery of YFP signal in the nucleus (Fig. 5F). Y2H assays further verified the interaction between CsZAT12 and CsNAC87, as yeast cells co-transformed with pGADT7-CsZAT12 and pGBKT7-CsNAC87 grew well on SD/−A/-H/−T/−L medium containing X-α-gal (Fig. 5G). In summary, these data strongly indicate that CsZAT12 physically interacts with CsNAC87.

To assess the effect of the CsZAT12-CsNAC87 interaction on *CsPORA* promoter activity, we measured the relative luminescence (LUC/REN ratio) using a dual-luciferase reporter system. The results showed that co-expression of *CsZAT12* and *CsNAC87* led to more pronounced suppression of *CsPORA* promoter-driven LUC activity compared to CsNAC87 alone (Fig. 5H). GUS staining results showed that co-expression of *CsZAT12* and *CsNAC87* exerted more stronger suppression than CsNAC87 on the GUS activity driven by the *CsPORA* promoter (Fig. 5I). Totally, these results demonstrate that CsZAT12 interacts with CsNAC87 to enhance the inhibition of *CsPORA* expression, resulting in the obstruction of chlorophyll biosynthesis in tea plants.

### CsZAT12 negatively regulates chlorophyll accumulation

**Figure 6:**
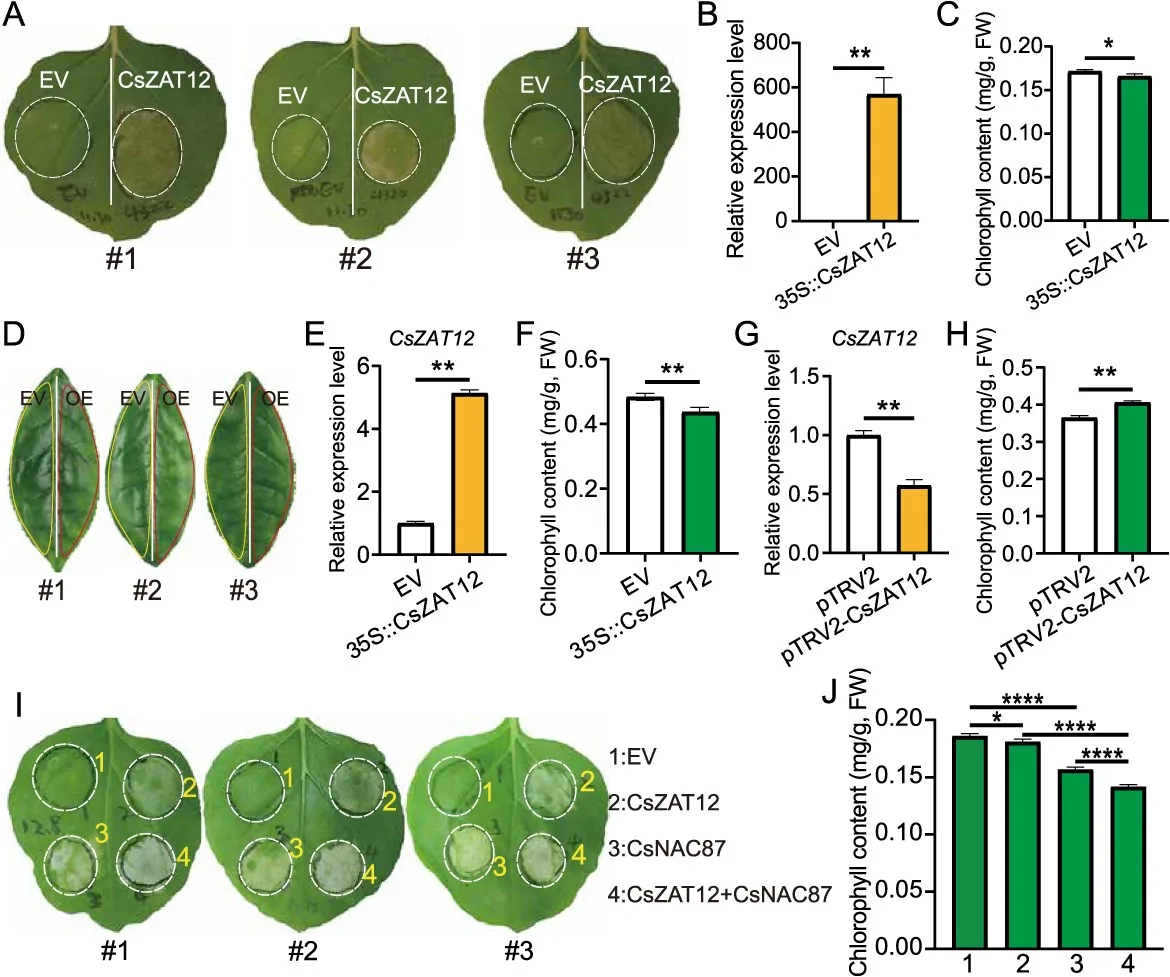
CsZAT12 negatively regulates chlorophyll accumulation. (A) Leaf color phenotype in *CsZAT12*-overexpressing tobacco leaves. (B) Relative expression of *CsZAT12* in transgenic tobacco leaves. (C) Determination of chlorophyll content in transgenic tobacco leaves of *CsZAT12*. (D) Leaf color phenotype in *CsZAT12*-overexpressing tea leaves. (E and G) Relative expression of *CsZAT12* in *CsZAT12*-overexpressing or silenced tea leaves. (F and H) Chlorophyll content in *CsZAT12*-overexpressing or silenced tea leaves. (I and J) Leaf color phenotype and total chlorophyll content in transgenic tobacco leaves of EV, *CsZAT12*, *CsNAC87* and the combination of *CsZAT12* and *CsNAC87*. (I–J) 1, EV; 2, 35S::CsZAT12; 3, 35S::CsNAC87; 4, 35S::CsZAT12 + 35S::CsNAC87.

To investigate the role of CsZAT12 in chlorophyll metabolism, we performed transient overexpression and silencing assays in tobacco and tea plants. *CsZAT12* overexpression in tobacco leaves achieved albino phenotype with its expression significantly upregulated (Fig. 6A and B). The content of total chlorophyll significantly declined (Fig. 6C). Overexpression of *CsZAT12* in tea leaves induced chlorosis phenotype (Fig. 6D and E). Compared to the control, the total chlorophyll content was notably reduced (Fig. 6F). *CsZAT12* silencing markedly decreased its expression (Fig. 6G), and chlorophyll content was significantly increased compared with the control (Fig. 6H). Furthermore, co-expression of *CsZAT12* and *CsNAC87* in tobacco leaves resulted in a more pronounced albino phenotype than that resulting from overexpression of *CsZAT12* or *CsNAC87* individually (Fig. 6I). The total chlorophyll content in tobacco leaves co-expressing both genes was significantly lower than that in leaves overexpressing either gene alone (Fig. 6J) These findings verify that CsZAT12 and CsNAC87 collaboratively suppress chlorophyll accumulation.

### Low temperature markedly induces *CsNAC87* and *CsZAT12* expression and reduces chlorophyll content

**Figure 7:**
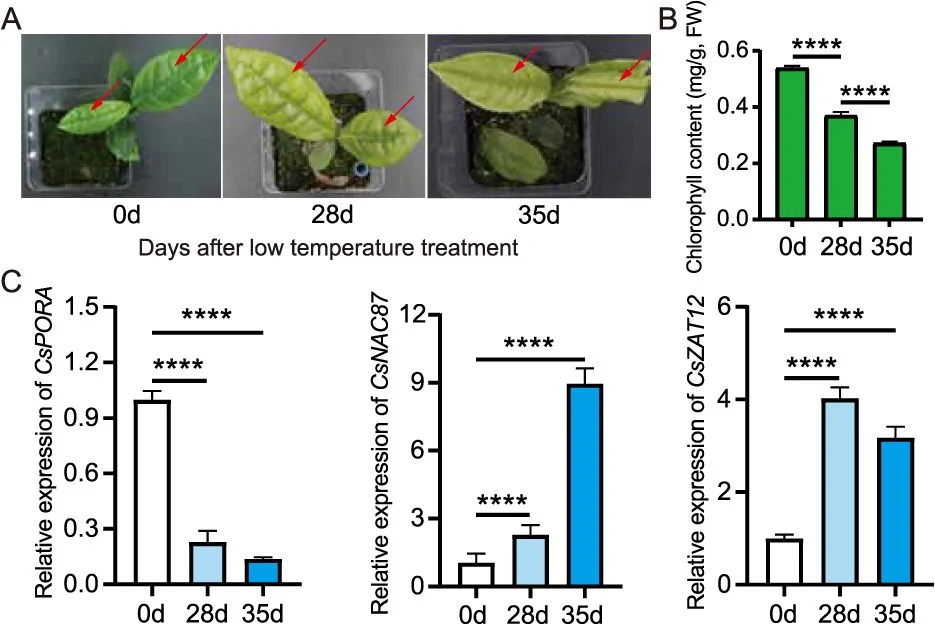
Low temperature treatment inhibits chlorophyll accumulation in ‘Baiye 1’ tea plants. (A) Phenotype of young leaves from cold-treated ‘Baiye 1’ cuttings. (B) Analysis of chlorophyll content in fresh leaves of ‘Baiye 1’ under cold conditions. (C) Expression pattern analysis of *CsPORA*, *CsNAC87*, and *CsZAT12* in response to low temperature. ‘d’ represents the days after low-temperature treatment.

To determine whether the expression of *CsPORA*, *CsNAC87*, and *CsZAT12* is regulated by low temperature, we subjected cuttings of ‘Baiye 1’ tea plants to low-temperature treatment. This treatment triggered leaf chlorosis, which progressed to a pronounced albino phenotype after 35 days (Fig. 7A). Chlorophyll content exhibited a substantial decrease following 28 and 35 days of cold exposure compared to the control (0 day) (Fig. 7B). Under low-temperature conditions, the expression of *CsPORA* was significantly down-regulated, while the expression of *CsNAC87* and *CsZAT12* was markedly up-regulated (Fig. 7C). In contrast, the young leaves of ‘Baiye 1’ at 25°C remained green with no significant changes in chlorophyll content or the expression of *CsPORA, CsZAT12*, and *CsNAC87* (Fig. S5). These results suggest that low temperature promotes the expression of *CsNAC87* and *CsZAT12* but represses *CsPORA* expression, thereby inhibiting chlorophyll accumulation and leading to chlorophyll deficiency in tender leaves of ‘Baiye 1’.

## Discussion

### Albino leaves of cold-sensitive tea plants are lack of chlorophyll

China is the primary origin of tea plants and possesses abundant tea germplasm resources. These rich resources provide ideal materials for investigating pigment metabolism. The rich diversity of leaf color among different tea accessions primarily results from the difference in pigment content, such as chlorophylls, carotenoids, and anthocyanins [3, 4, 6]. It is well known that the albino tea plant is not only famous for its albino leaves, but also for its rich free amino acids accumulated in albino leaves. However, the regulation of chlorophyll metabolism in albino tea leaves is still largely unknown.

The content of pigments in albino tea leaves is greatly affected by low temperature. For instance, exposure to a low temperature (15°C) induced an albino leaf phenotype and reduced chlorophyll content in the cold-sensitive albino tea cultivar ‘Xiaoxueya’ [7]. In the cold-sensitive albino tea cultivar ‘Huabai 1’, exposure to 15°C induced leaf albinism and reduced chlorophyll content [2, 8]. Here, low-temperature treatment at 15°C induced albino leaves in ‘Baiye 1’ along with significant reduction in chlorophyll content (Fig. 7A and B), while fresh leaves of ‘Baiye 1’ remained green with no significant changes in chlorophyll content under normal conditions (Fig. S5). Similar result was also achieved in Xu’s research [6]. Notably, regardless of whether the temperature was set at 15°C, 25°C, or 30°C, the tender leaves of ‘Longjing 43’ and ‘Zhonghuang 1’ tea plants showed no signs of albinism [6]. These results indicate that ‘Baiye 1’ tends to bleaching under low temperature conditions. Similar phenotype has been observed in cold-induced albino rice [27]. Moreover, the leaf color transition in tender leaves of ‘Baiye 1’ was highly correlated with chlorophyll content (Fig. 1). This correlation aligns with previous findings that leaf color corresponds to chlorophyll level in tender leaves of ‘Baiye 1’ [28]. Collectively, these observations underscore that a substantial reduction in chlorophyll content is a fundamental factor of the albino leaf trait in tea plants.

### Pronounced downregulation of *CsPORA* contributes to the albino leaf phenotype of tea plants

Although the chlorophyll metabolic pathway in albino tea leaves has been well-documented [5, 6, 29], most studies have focused primarily on gene expression analyses. To bridge this gap, we quantified chlorophyll content in tender leaves of ‘Baiye 1’, which exhibited different color phenotypes. After integrating with RNA-seq data, we identified eight differentially expressed chlorophyll metabolism-related genes, including *CsCHLH*, *CsCLH2.1*, *CsCLH2.2*, *CsNYC1*, *CsHEM6*, *CsHEM11*, *CsCAO*, and *CsPORA* (Fig. 2D and E). *CsNYC1* (*CSS0034731*) regulates chlorophyll deficiency in yellow-leaved ‘Nanchuan Huangye 1’ tea plants [15]. Here, the expression of *CsNYC1* was lower in albino tea leaves than that in green leaves (Fig. 2E). This discrepancy suggests that *CsNYC1* is unlikely to be the primary regulator of chlorophyll deficiency in albino tea leaves. In contrast, *CsPORA* exhibited a strong positive correlation with chlorophyll content (Fig. 2E**,**  Fig. S1). Previous research revealed that *CsPOR1* expressed lowly in albino tea leaves [30]. Moreover, silencing of *CsPOR1* (CSS0033593) induced photo-bleached tea leaves [13]. However, the expression of *CsPOR1* did not differentially expressed between albino tea leaves and green leaves (S1 vs S2; S2 vs S7). Compared to green leaves, the expression of *CsPORA* is significantly lower in albino tea leaves of ‘Baiye 1’ (Fig. 2E; Fig. 7A and C) and ‘Huangshanbaicha’ tea plants [29]. Low temperature treatment markedly decreased *CsPORA* expression and chlorophyll content (Fig. 7). Both Overexpression and silencing of *CsPORA* proved its role in positively regulating chlorophyll accumulation. Thus, these results suggest that the significant reduction of *CsPORA* in albino tea leaves may be an important factor contributing to the albino leaf trait of ‘Baiye 1’. A recent study proved that the magnesium chelatase enzyme subunit CsCHLI^400R^ is responsible for the yellow-leaved trait of light-sensitive tea cultivar ‘Baijiguan’ [4]. To investigate whether CsCHLI is the key regulator in albino tea leaves, we determined the genotype of CsCHLI in ‘Baiye 1’. The genotype of ‘Baiye 1’ was identified as CsCHLI^400H^ (Fig. S6). This result indicates that CsCHLI does not regulate the albino leaf trait in tea plants. Overall, our results suggest that the chlorophyll deficiency in albino tea leaves may result from a block in chlorophyll synthesis.

### CsNAC87 negatively regulates chlorophyll accumulation via inhibiting *CsPORA* expression

NAC transcription factors are pivotal regulators of chlorophyll metabolism, senescence processes and cold stress responses in plants [17, 31, 32]. However, the NAC-mediated chlorophyll metabolism in tea plants remains unexplored.

In this study, we identified CsNAC87 as a cold-responsive regulator of chlorophyll metabolism in albino tea leaves. *CsNAC87* was highly expressed in albino tea leaves but expressed at low levels in green leaves (Fig. 1; Fig. 5A), and its expression was induced by low temperature (Fig. 7C). CsNAC87 directly represses *CsPORA* expression by binding to the TTACTTGT motif in its promoter, thereby negatively regulating chlorophyll accumulation (Fig. 4). These results highlight the important role of CsNAC87 in cold-induced leaf albinism of tea plants. In *Arabidopsis*, *ANAC087* overexpression accelerates chlorophyll catabolic metabolism, programmed cell death, and reactive oxygen species (ROS) levels to promote leaf senescence [17]. Here, overexpression of *CsNAC87* also enhances ROS levels and chlorophyll degradation in tobacco (Fig. S7; Fig. 4E–G). Excessive accumulation of ROS leads to damage to the antioxidant enzyme system, which triggers membrane lipid peroxidation and accelerates the decomposition of chlorophyll, disrupting the stability of chloroplasts [33]. In a cold-sensitive tea variety ‘Yinghong 9’, the expression of ROS metabolic enzymes (CsAPX2, CsGST2, and CsSOD2) significantly changes under cold stress, accompanied by relatively distinct symptoms of chloroplast damage [34]. Moreover, cold stress promotes ROS accumulation leading to leaf albinism or etiolation in rice [27] and cucumber [24]. We thereby speculate that CsNAC87 not only inhibits chlorophyll accumulation by repressing *CsPORA* expression, but may also function by increasing ROS levels. This functional profile appears distinct from some NAC homologs. PpNAP4 in peach concurrently promotes chlorophyll degradation and anthocyanin biosynthesis via activating *PpSGR*, *PpPAO*, *PpPPH*, *PpANS*, and *PpMYB10*.*1* [35]. LcNAC002 regulates chlorophyll degradation in litchi by activating *LcSGR* expression [36]. Notably, NAC87 orthologs in other species modulate hormone pathways, such as gibberellin [18] and JA-mediated senescence [37]. Thus, it indicates that CsNAC87 may contribute to regulating leaf albinism in tea plants through a network involving chlorophyll metabolism, ROS modulation, and potential hormone signaling. Further research is warranted to dissect these interconnected pathways underlying CsNAC87’s role in tea plants.

### Cold-responsive CsZAT12-CsNAC87 module represses chlorophyll accumulation in albino tea leaves

Intriguingly, we identified a C2H2-type zinc finger protein CsZAT12 in albino leaves of ‘Baiye 1’ whose expression exhibiting high correlation with *CsNAC87* (Fig. 5A–C). *CsZAT12* overexpression decreased chlorophyll content in tobacco leaves (Fig. 6A–C) and tea plants (Fig. 6D–F). In contrast, SlZAT5 in tomato positively regulates chlorophyll content to delay fruit ripening [21]. In tea plants, CsMYB308 interacts with CsDOF3 to inhibit chlorophyll accumulation by activating *CsCLH1* expression [16]. Here, CsZAT12 physically interacts with CsNAC87 and enhances its repressive effect on *CsPORA* (Fig. 5D–I). This synergistic effect was phenotypically evident, as co-expression of *CsNAC87* and *CsZAT12* in tobacco leaves resulted in a complete albino phenotype with a more severe chlorophyll deficiency than that resulting from overexpressing *CsZAT12* or *CsNAC87* alone (Fig. 6I and J). Low temperature induced the expression of *CsZAT12* and *CsNAC87* (Fig. 7C), and overexpression of *CsZAT12* and *CsNAC87* promoted the accumulation of ROS in tobacco (Fig. S7). The *SGR* gene acts as a ‘switch’ in the chlorophyll degradation pathway, and its overexpression promotes ROS accumulation in chloroplasts, which accelerates chlorophyll degradation and cell death [33]. In turn, the ROS signal also upregulates *SGR*, *NYC1* and *PAO* expression [38]. Thus, these findings suggest that CsZAT12 and CsNAC87 may activate chlorophyll catabolism. Considering that the biological functions of CsZAT12 and CsNAC87 in chlorophyll metabolism were studied via transient assays in this study, further investigation of their potential functions (e.g. chlorophyll catabolism, hormone metabolism) via constructing stable transgenic lines is warranted. In contrast, a zinc finger protein PvSSG interacts with PvNAP1/2 to inhibit the DNA binding ability of PvNAP1/2 in the promoters of *PvNOL*, *PvSGR*, and *PvPAO*, impeding chlorophyll degradation and delaying leaf senescence in switchgrass [39]. BnaNAC87 positively regulates *BnaZAT12* to modulate ROS accumulation and cell death, repressing chlorophyll content in *Brassica napus* [40]. These comparisons underscore the functional diversity of ZATs and NACs in chlorophyll metabolism in different species. Collectively, our work unveils a novel cold-responsive CsZAT12-CsNAC87 module as an important regulator of chlorophyll metabolism in tea plants.

**Figure 8:**
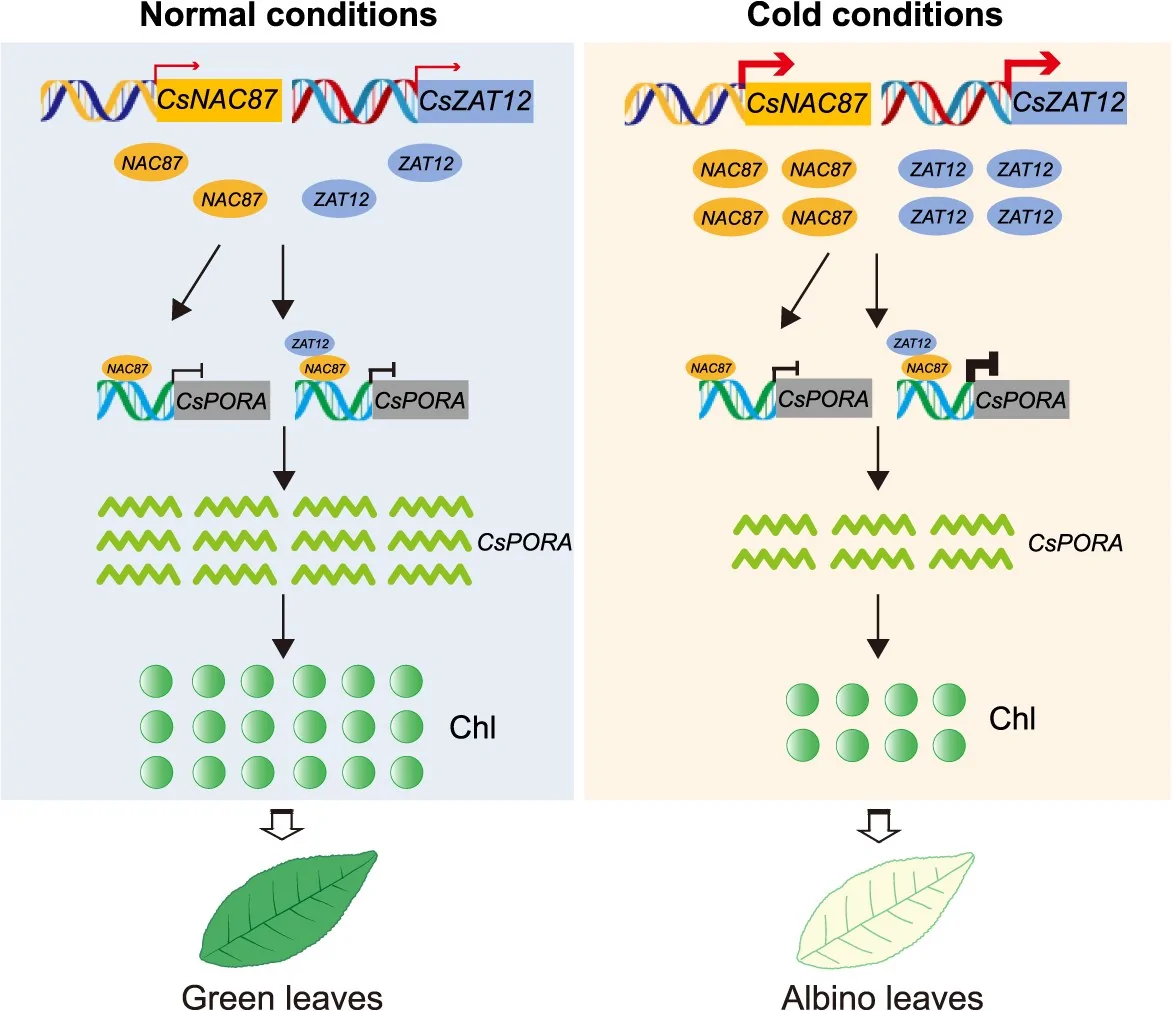
A proposed model of CsZAT12-CsNAC87 module regulate chlorophyll metabolism in tea plants via mediating *CsPORA* expression.

In summary, we propose a model illustrating the modulation of chlorophyll metabolism, leading to either green or albino leaves in tea plants (Fig. 8). Under normal conditions, the low expression levels of both *CsZAT12* and *CsNAC87* limit their interaction, thereby attenuating the repression of *CsPORA* expression by CsNAC87. Under cold conditions, the expressions of *CsZAT12* and *CsNAC87* are induced. The interaction between CsZAT12 and CsNAC87 enhances the transcriptional repression of *CsPORA*, resulting in reduced chlorophyll accumulation, and thus the tea leaves turn white. This model posits that the CsZAT12-CsNAC87 module acts as an important cold-responsive switch, regulating chlorophyll metabolism in tea leaves by modulating *CsPORA* expression. These findings advance our understanding of chlorophyll metabolism in albino tea leaves.

## Materials and methods

### Plant materials

‘Baiye 1’ tea plants were cultivated in Anji Country, Zhejiang, China. Tender leaves (one bud and one leaf) were collected during seven developmental periods (S1: March 28, S2: April 6, S3: April 12, S4: April 20, S5: April 27, S6: May 4, S7: May 16) in 2022. After harvesting, samples were flash-frozen in liquid nitrogen and stored at −80°C. Cuttings of ‘Baiye 1’ tea plants were cultivated in the greenhouse. Five-week-old *N. benthamiana* plants served as transient expression hosts.

### Low-temperature treatment assays

To investigate the effect of cold conditions on chlorophyll metabolism and the expression of *CsPORA*, *CsZAT12*, and *CsNAC87*, one-year-old cuttings of ‘Baiye 1’ were subjected to low temperature treatment as described in previous study [6]. Cuttings of ‘Baiye 1’ were grown in a light incubator at 15°C/10°C (day/night); the control condition was 25°C/25°C, with 600 μmol m^−2^·s^−1^ PAR, a 16 h/8 h photoperiod, and 65% relative humidity. The first and second leaves of ‘Baiye 1’ tea plants were sampled after 0, 28, and 35 days of low temperature treatment. Each treatment included at least three independent biological replicates. These samples were prepared for gene expression analysis and chlorophyll content determination.

### RNA extraction and cDNA synthesis

Total RNA was isolated with the FastPure® Plant RNA Kit according to the manufacturer’s protocol. RNA integrity was assessed by agarose gel electrophoresis, and its concentration was determined using a NanoDrop 2000 spectrophotometer. The HiScript® III 1st Strand cDNA Synthesis Kit (with gDNA wiper) was used to synthesize cDNA from total RNA.

### Chlorophyll content measurement

Chlorophyll contents in tea and tobacco leaves were measured using Lichtenthaler’s method [41]. Sample powder (50 mg) was homogenized in 0.5 ml ice-cold 80% acetone. After centrifugation (12 000 × g, 15 min, 4°C), the supernatant absorbance was measured at 646 and 663 nm using a SpectraMax M2 microplate reader. The content of chlorophyll was calculated accordingly. Chlorophyll a (Chl *a*) = [(12.25 × A_663_–2.79 × A_646_) × V]/(W × 1000); Chlorophyll b (Chl *b*) = [(21.50 × A_646_–5.10 × A_663_) × V]/(W × 1000). The experiment was performed with at least three replicates.

### Antisense oligonucleotide in tea leaves

Antisense oligonucleotides (AsODN) and sense oligonucleotides (sODN) targeting *CsPORA* were designed using Soligo (https://sfold.wadsworth.org/) and synthesized by Youkang Biotech (Table S3). For gene silencing, a 20 μM solution containing both AsODN and sODN solutions was infiltrated into tea leaves. Samples were harvested after 24 h and prepared for metabolite and gene expression analyses.

### Transient overexpression assay

Coding sequences (CDS) of *CsPORA*, *CsZAT12*, and *CsNAC87* were amplified from albino leaves of ‘Baiye 1’ and cloned into pCAMBIA1305, respectively (Table S4). Recombinant plasmids were transformed into Agrobacterium GV3101. Transient overexpression in tobacco leaves was performed as described below. Positive strains were cultured and then resuspended in MMA buffer (10 mM MgCl_2_, 10 mM MES, 100 μM acetosyringone (AS), pH 5.6). After incubating for two hours in the dark, the inoculation solution was infiltrated into tobacco leaves. EV served as the control. After four days, leaves were harvested for DAB staining, metabolite content determination, and gene expression analyses.

Transient overexpression in tea leaves was performed followed Chen’s method [42] with minor modifications. Positive strains harboring pCAMBIA1305-CsZAT12 or pCAMBIA1305-CsNAC87 were cultured and then resuspended in MMA buffer. pCAMBIA1305 vector was the negative control. Four days later, samples were harvested for gene expression and metabolite analyses.

### Virus-induced gene silencing assay in tea leaves

We performed virus-induced gene silencing (VIGS) assay in tea leaves using the method described by Gao with minor modifications [43]. Target-specific 300 bp CDS fragment of *CsZAT12* was inserted into pTRV2 (Table S4), with pTRV1 as helper plasmid. Recombinant vectors were transformed into Agrobacterium GV3101, cultured to OD600 = 1.0 in the MMA buffer. Equal-volumes of mixtures (pTRV1 + pTRV2; pTRV1 + pTRV2-CsZAT12) were infiltrated into mature leaves of ‘Baiye 1’. After 24-hour dark incubation and eight-day greenhouse growth, silenced leaves were collected for gene expression analyses and metabolite content determination.

### Y1H assay

Yeast one-hybrid (Y1H) was performed using the Matchmaker One-Hybrid system. The CDS of *CsNAC87* was inserted into the pGADT7 vector, and the promoter fragment of *CsPORA* was inserted into the pAbAi vector (Table S4). Bait vector integration into Y1HGold cells was confirmed before prey vector co-transformation. Empty vector controls were included. Transformants were selected on SD/−Leu medium [without Leu]. After three days of culture at 30°C, the positive clones were further cultured on SD/−Leu containing AbA (Aureobasidin A).

### Dual luciferase assay

Dual LUC assay was conducted as follows. Effector-reporter system was constructed with p1305-CsNAC87, and the promoter of *CsPORA* was cloned into pGreenII0800 (Table S4). Recombinant plasmids were transformed into *A. tumefaciens* GV3101 (pSoup-p19). Positive strains were cultured and resuspended in MMA buffer. Effector-reporter mixtures were infiltrated into tobacco leaves. Three days later, infiltrated leaves were treated using 150 μg/ml D-luciferin. Luminescence signal was quantified using NightSHADE LB 985 N with LUC/REN ratios determined by dual-luciferase assays.

### Electrophoretic mobility shift assay

The CDS of CsNAC87 was cloned into the pMAL-C2X vector. The purified MBP and MBP-CsNAC87 proteins were incubated with biotin-labeled probe (TTTTATATGATTACTTGTATAAATTCGTA) in binding buffer. For the competition assay, cold competitors were added to the reaction to detect the binding of CsNAC87 to the promoter of *CsPORA*. These samples were incubated at 25°C for 30 min. Then the reaction mixtures were separated by a 6% native polyacrylamide gel and transferred to a nylon membrane. The following steps were carried out according to the instructions of the Chemiluminescent EMSA Kit (Beyotime, Shanghai, China). Luminescence intensity was captured by a Gel Imaging System.

### GUS analysis

The promoter of *CsPORA* was cloned into the pCAMBIA1304 vector. Positive Agrobacterium strains harboring the expression vectors (pCAMBIA1305-CsZAT12 and pCAMBIA1305-CsNAC87) were separately infiltrated into tobacco leaves. Three days later, infiltrated tobacco leaves were collected for GUS staining. GUS staining assay was performed using a GUS Staining Kit (Coolaber, Beijing, China) according to the manufacturer’s instructions.

### Yeast two-hybrid assay

Yeast two-hybrid (Y2H) assays were conducted according to the manufacturer’s protocol. Specifically, the CDS of *CsNAC87* and *CsZAT12* were cloned into pGBKT7 (bait vector) and pGADT7 (prey vector), respectively (Table S4). Recombinant plasmids were co-transformed into yeast strain Y2HGold. Transformants were selected on SD medium without leucine (Leu) and tryptophan (Trp) (SD-L/−T). Yeast cells were selected on SD medium without trp, leu, His, and adenine (Ade) (SD-T/−L/-H/−A) with or without AbA and X-α-gal. Appropriate controls were included: pGBKT7–53/pGADT7-T served as a positive control, while pGBKT7-Lam/pGADT7 was used as a negative control.

### Bimolecular fluorescence complementation assay

BiFC assays were conducted as described below. The CDS of *CsZAT12* and *CsNAC87* were cloned into pCNHP-cEYFP and pCNHP-nEYFP to fuse cEYFP and nEYFP fragments, respectively (Table S4). Constructs (CsZAT12-cEYFP and nEYFP-CsNAC87) were separately transformed into Agrobacterium GV3101 (pSoup-p19), and the resulting strains were co-infiltrated into tobacco leaves. After three days, fluorescence was imaged using a confocal microscope with excitation/emission settings: 514 nm/540–570 nm (EYFP); 545 nm/590–610 nm (mCherry).

### Firefly luciferase complementation imaging assay

Firefly LCI assays were conducted as follows. The CDS of *CsZAT12* and *CsNAC87* were cloned in-frame into nLUC (JW-771) and cLUC (JW-772) vectors, respectively (Table S4). Constructs were transformed into Agrobacterium GV3101 and equal-volume suspensions (CsNAC87-cLUC and CsZAT12-nLUC) were co-infiltrated into tobacco leaves. After three days, leaves were sprayed with 150 μg/ml luciferin and imaged using a NightSHADE LB 985 N system.

### Co-immunoprecipitation

Co-immunoprecipitation (Co-IP) assays were performed as follows. Briefly, recombinant vectors CsZAT12-GFP (pCAMBIA1305-CsZAT12) and CsNAC87–3 × Flag (pK4.1-CsNAC87) were constructed and transformed into *Agrobacterium*. Positive strains were infiltrated into tobacco leaves. The control groups comprised tobacco leaves co-infiltrated with CsZAT12-GFP and empty 3 × Flag vector, and CsNAC87–3 × Flag and empty GFP vector. The experimental group was co-infiltrated with CsZAT12-GFP and CsNAC87–3 × Flag. Three days post-infiltration, total protein was extracted from the infiltrated leaves. The protein extracts were individually incubated with anti-GFP beads at 4°C for 10 h. After washing with prechilled TBST buffer, the beads were boiled in SDS loading buffer for 10 min. The co-precipitated proteins were analyzed by western blotting. An anti-Flag antibody was used to detect CsNAC87–3 × Flag and assess its co-precipitation with CsZAT12-GFP. An anti-GFP antibody was used to determine whether CsZAT12-GFP and GFP were expressed. An anti-Actin antibody served as a control to ensure equal sample input.

### Quantitative real-time PCR

Gene expression was analyzed by quantitative real-time PCR with *CsGAPDH* and *NtGAPDH* as reference genes. Relative expression levels were calculated using the 2^−ΔΔCt^ method [44]. Three biological replicates were included for each sample. Primer sequences are listed in Table S4.

### Statistical analysis

Transcriptome data [45] of ‘Baiye 1’ were used for DEG analysis. DEGs (log_2_|fold change| ≥ 1) were visualized via volcano plots (http://www.bioinformatics.com.cn). GO and KEGG enrichment analyses were performed using ggplot2 in R. Gene expression profiles were analyzed with TBtools [46]. One-way analysis of variance (ANOVA) was used for statistical analysis. Statistically significant differences are indicated with ^*^P < 0.05, ^**^P < 0.01, ^***^P < 0.001, ^****^P < 0.0001. Data are expressed as mean ± SD (n ≥ 3).

## Supplementary Material

Web_Material_uhag295

## Data Availability

The data underlying this article are available in the article and in its online supplementary material.
