# Supplementary material for: CsZAT12 interacts with CsNAC87 to repress chlorophyll accumulation in albino leaves of tea plants
Source: Hortic Res. 2026 Jul 9;13(9):uhag295. doi: 10.1093/hr/uhag295 (PMC13557926; doi:10.1093/hr/uhag295)
Supplement: Supplementary file 1 [file web_material_uhag295.zip › Supplemental figures.docx]

**Figure S1. Correlation analyses of eight chlorophyll metabolism-related genes with chlorophyll content in ‘Baiye 1’ across different periods**.

**Figure S2.** **Correlation analyses of *CsPORA* with the transcription factors identified from DEGs.** (**A**) Correlation network analyses of *CsPORA* with the transcription factors from DEGs. (**B**) Correlation analysis of *CsPORA* with the expression of CsNAC87 across different periods. (**C**) Correlation analysis of chlorophyll content (Chl *a* + Chl *b*) with the expression of CsNAC87 across different periods.

**Figure S3. SDS‑PAGE analysis of MBP‑CsNAC87 protein expressed in *E. coli*.** Lane 2: protein molecular weight marker (with sizes indicated). Lane6: total induced protein of MBP‑CsNAC87 before ultrasonication. Lane7: soluble fraction (supernatant) of induced protein after ultrasonication. Lane8: insoluble fraction (precipitation) of induced protein after ultrasonication. Lane9: purified MBP‑CsNAC87 protein eluted from MBP resin.

**Figure S4. Western blot analysis of the interaction between CsZAT12-GFP and CsNAC87-Flag in tobacco leaves.** (A) Reciprocal Co-immunoprecipitation (Co-IP) detected with anti-Flag antibody; (B) An anti-GFP antibody to detect CsZAT12-GFP; (C) Co-IP detected with anti-Flag antibody (for CsNAC87-Flag); (D) An anti-Actin antibody as a loading control. Lanes L1, L2, and L3 represent the following sample groups: L1, p1305-CsZAT12 + pK4.1 (empty vector); L2, p1305 (empty vector) + pK4.1-CsNAC87-Flag; L3, p1305-CsZAT12 + pK4.1-CsNAC87-Flag.

**Figure S5.** **Chlorophyll accumulation and gene expression profiles in ‘Baiye 1’ cuttings grown at ambient temperature**. (**A**) Phenotype of ‘Baiye 1’ cuttings under ambient temperature treatment (25°C). (**B**) Chlorophyll content in fresh leaves of ‘Baiye 1’. (**C**) Expression pattern of *CsPORA*, *CsNAC87*, and *CsZAT12* in ‘Baiye 1’. ‘d’ represents the days after ambient temperature treatment. CK represents the group of ‘Baiye 1’ under ambient temperature (25°C) treatment.

**Figure S6.** **Partial sequence alignment of CsCHLI from ‘Baiye 1’ and ‘Baijiguan’ tea cultivars**. (**A**) Coding sequences alignment of *CsCHLI*. (**B**) Amino acid sequences alignment of CsCHLI.

**Figure S7. O****verexpression of *CsZAT12* and *CsNAC87* induces the accumulation of ROS**. DAB staining of transgenic tobacco leaves of *CsZAT12* (A) and *CsNAC87* (B).
